# Land use affects lowland stream ecosystems through dissolved oxygen regimes

**DOI:** 10.1038/s41598-019-56046-1

**Published:** 2019-12-23

**Authors:** Paula C. dos Reis Oliveira, Harm G. van der Geest, Michiel H. S. Kraak, Piet F. M. Verdonschot

**Affiliations:** 1 Department of Freshwater and Marine Ecology, Institute for Biodiversity and Ecosystem Dynamics, University of Amsterdam, P.O. Box 94248, 1090 GE Amsterdam, The Netherlands; 2 Freshwater Ecology Group, Wageningen Environmental Research, Wageningen UR, P.O. Box 47, 6700 AA Wageningen, The Netherlands

**Keywords:** Freshwater ecology, Ecosystem ecology, Limnology

## Abstract

The aim of the present study was to assess the impact of surrounding land use on the structure and functioning of lowland stream ecosystems. To this end, five different land use types were selected (forest, extensive grassland, intensive grassland, cropland and wastewater treatment plant) each represented by four replicate streams, in which diel dissolved oxygen concentrations were recorded, sediment and water quality parameters were measured and macroinvertebrate community composition was determined. *Chironomus* sp., Oligochaeta and Gastropoda dominated the cropland and wastewater treatment plant (WWTP) streams, while Plecoptera and most Trichoptera only occurred in forest and extensive grassland streams. Forest streams communities were related to a high oxygen saturation, a high C/N ratio in the sediment and woody debris and coarse particulate organic matter (CPOM) substrate cover. Macroinvertebrate communities in cropland and WWTP streams were related to a low oxygen saturation in water and sediment and high concentrations of dissolved nitrogen, phosphorus and carbon. It is concluded that land use specific impacts on lowland streams are likely exerted via fine sediment accumulation in deposition zones, affecting oxygen regimes, sediment oxygen demand and stream metabolism, ultimately changing macroinvertebrate community composition. This study supports therefore the importance of including the catchment scale in ecological stream quality assessments, combining structural and functional endpoints.

## Introduction

The ecological structure and functioning of streams strongly depends on the surrounding land use^1^. The most studied impacts of land use type on stream ecosystems include effects on hydromorphology^2^, water quality^3^ and riparian habitats^4^. These studies mainly focussed on high gradient streams, but also in lowland streams, flowing through alluvial plains that are historically impacted by agriculture and urbanization, a strong effect of land use type on stream ecosystems may be expected.

One of the most important stressors originating from the adjacent land is the input of fine sediments into streams^5^. These fine sediments accumulate in deposition zones, like stream bed depressions and pools^6^, especially in streams with low current velocities such as lowland streams^7^. Fine sediments increase the turbidity^8^, decrease the underwater light availability for primary producers^9^ and reduce the availability of suitable streambed habitats for aquatic invertebrates^10^. In addition, changes in the physicochemical conditions of the streambed may lead to changes in nutrient dynamics^11^, oxygen concentrations^12^ and biofilm assemblages^13–15^.

Since the type of fine sediments entering the streams is land use type specific, the subsequent effects on the ecological structure and functioning of streams are expected to be land use type specific as well^16,17^. Indeed, the effects of changes in dissolved oxygen concentrations resulting from fine sediment inputs on macroinvertebrate community composition are well documented^3,16,18^. However, the mechanisms underlying these land use type specific effects of fine sediment inputs remain poorly studied, especially in lowland streams.

The aim of the present study was therefore to assess the impact of land use type specific inputs of fine sediment on the structure and functioning of lowland stream ecosystems. We based our study on three complementary pillars by assessing water and sediment physico-chemical characteristics, stream metabolism and macroinvertebrate community composition. We hypothesised that different land use types result in differences in substrate composition and related physicochemical characteristics of stream deposition zones. These changes in streambed characteristics were expected to affect stream metabolism and therewith diel dissolved oxygen regimes, which in turn affect macroinvertebrate communities. To test this hypothesis, five different land use types were selected, each represented by four replicate streams in which diel dissolved oxygen concentrations were recorded, sediment and water quality parameters were measured and macroinvertebrate community composition was determined.

## Materials and Methods

### Study sites

The present study was conducted under late summer conditions from September 20^th^ until October 15^th^ 2017, in twenty lowland streams in the Netherlands representing five common land use types (Supplementary Table S1). For each land use type, four replicate streams with similar morphological characteristics were selected (Supplementary Table S1 and Fig. S1). The land use types included forest areas, serving as natural reference sites (hereafter referred to as forest), non-fertilized pasture (extensive grassland (EG)), fertilized pasture (intensive grassland (IG)), arable field (cropland) and wastewater treatment plants, releasing effluent about every 15 minutes (WWTP). The selection criteria for the streams in the forest, grassland and cropland catchments was based on the percentage of surface covered (>2/3) by the selected land use type, as indicated on the national Dutch land use map (LGN5)^19^ (Fig. S1). WWTP streams were selected based on the presence of a sewage treatment plant (~50.000 people) outflow, no more than 250 m away from the selected stretch in each of the replicate streams.

Stream width and depth were measured in a 20 m stretch in each studied stream. Mean width was calculated by measuring width every 2 m. Mean stream depth and current velocity (electromagnetic current meter Valeport model 802) were measured and discharge was calculated at five sites equally distributed along three transects (upstream, middle and downstream) along the stream stretch.

The field experiment lasted 48 h at each site, but because of logistic limitations, 25 days in total were needed to run the entire study. Each time that the 48 h oxygen measurements in the field were completed, water, sediment and invertebrate samples were taken before the field experiment was uninstalled. Below the corresponding methods are described in more detail.

### Stream water chemistry

In each stream, a water sample (1 L) was collected in plastic bottles and stored at −20 °C. After thawing, subsamples were taken for the following measurements. Conductivity and pH were measured from a subsample of about 50 ml in a glass vial with a portable multi sensor meter (HQ440d HACH), and a 15 ml subsample was taken to measure turbidity (Hach 2100Q meter) in the laboratory at 20 °C. A filtered (0.2 µm GFC filter) 10 ml subsample was taken in polystyrene tubes for ammonium (NH4^+^), nitrate (NO_3_^−^), nitrite (NO_2_^−^) and phosphorus (PO_4_^3−^) measurements, analysed with a Skalar SAN++ segmented flow analyser packed with a 1074 twin needle autosampler, and software Flow Access v3.

### Sediment characteristics and substrate cover

In each stream deposition zones were identified, defined as deeper areas where current velocity was lower, measured with an electromagnetic current meter (Valeport model 802) and where fine particulate organic matter (FPOM) accumulated, identified according to Hering *et al*.^20^. In each stream, a composite sediment sample was taken from representative deposition zones by sampling the top 2 cm layer using an acrylic core. All samples were freeze-dried (CoolSafe 55-9 Pro) directly after sampling and subsequently analysed for sediment characteristics. Grain size distribution (Phi) was measured according to NEN 5753 (2006)^21^ and analysed following Wentworth^22^ and Blott and Pye^23^. Per stream a sediment subsample was ball-milled for organic matter content (OM), total carbon (TC), total nitrogen (TN), total phosphorus (TP), organic phosphorus (OP), inorganic phosphorus (IP) concentrations and chlorophyll-*a* content (Chl*a*) measurements. TC and TN were measured using an elemental analyzer (Elementar Vario EL, Hanau, Germany) and OM by loss of weight-on ignition of oven dried (105 °C) material at 550 °C for 16 hours. TP was determined by first weighing 2 sets of roughly 0.80 g of ball-milled sediment per sample and igniting one of the duplicate samples at 500 °C for 16 hours. Afterwards, both burnt and unburned samples were extracted using 0.5M of sulfuric acid from which the total phosphorus content was determined according to Murphy and Riley^24^. Inorganic phosphorus (IP) corresponded to the phosphorus fraction determined from unburned samples^24^. Organic phosphorus (OP) was calculated by subtracting inorganic from total phosphorus. Sediment chlorophyll–a concentrations were quantified according to Porra *et. al*.^25^ and Brito *et al*.^26^ and the respective concentrations were calculated using Lorenzen’s equation^27^, modified for sediment samples. In each stream, substrate cover was estimated according to Hering *et al*.^20^ in a twenty m stream stretch.

### Dissolved oxygen regime

In each stream, six Hobo U26-001 oxygen probes (Onset Computer Corporation) were installed just above the stream bed for continuous oxygen concentration and temperature measurements every five minutes over a period of 48 h, as recommended by Siders^28^ and Bott^29^. Two replicate probes were installed twenty meters apart in the main stream, while four replicate probes were installed at the deposition zones (Supplementary Fig. S2). Oxygen probes were installed in the four replicate streams of a given land use type at the same day. After two days they were moved to the next land use type until all twenty streams were measured. Before installation in the field, all oxygen probes were calibrated in the laboratory using the air-saturation water approach according to the calibration tool in HOBOware®. After retrieval, the probes were checked again in the laboratory by placing all probes at the same time in 100% saturated water for 3 hours. Three days prior to the installation of the oxygen probes, acrylic plates (2 m length; 0.5 m height; 3 mm width) were installed parallel to the main flow path to better separate deposition zones from the main flow path. Small patches of macrophytes were removed when growing too close to the probe. In addition, to avoid drifting macrophyte parts and filamentous algae accumulating on the oxygen sensors, bamboo sticks were placed 30–50 cm upstream of the probes. To avoid variation in the oxygen regime measurement due to temperature differences during the 25 days of the field experiment, dissolved oxygen concentrations were converted to oxygen saturation percentages correcting for water temperature according to Wetzel & Likens^30^. From the oxygen time series, the daily fluctuations and average oxygen concentration per stream per land use type were analysed. In addition, cumulative frequency distributions of the oxygen saturation classes from zero to 180 percent (in steps of 5%) were calculated.

### Sediment oxygen demand

For the measurements of the sediment oxygen demand (SOD), four replicate undisturbed sediment acrylic cores (6 cm diameter) per stream were taken by coring the top 10 cm of the sediment in the deposition zones. The remaining headspace of the cores was filled with water collected at the sites. The sediment cores were kept at 20 °C in the dark (covered with aluminium foil) and the overlaying water was saturated with air immediately after sampling and closed. Next, dissolved oxygen concentrations were measured directly, after 24 hours and at least two more times during these 24 hours with a multi-channel fibre optic meter (Oxy-4 *PreSens* Precision Sensing GmbH, Regensburg, Germany). From the decline in oxygen concentration over time, SOD was calculated according to Rong *et al*.^31^.

### Stream metabolism

To estimate stream metabolism, oxygen measurements were complemented with continuous (every 5 minutes) light intensity measurements using 1 HOBO Pendant™ probe per stream, installed next to one of the oxygen probes. The 48 hours continuous measurements of oxygen concentrations in mg/l, temperature and light intensities were entered into a Bayesian Single-Station Estimation model (BASE)^32^, to simultaneously estimate gross primary production, ecosystem respiration and the reaeration coefficient K (R-package BASEmetab, version 3).

We used one bar atmospheric pressure (sea level), zero salinity (freshwater) and the measured light intensity^33^ for the 1-station models to estimate gross primary production (GPP), ecosystem respiration (ER) and the reaeration coefficient K. GPP and ER were adjusted for mean stream depth. The default options of the BASEmetab package were used, including the assumptions described by Grace *et al*.^32^ and Song *et al*.^33^ R-squared values were used to assess the quality of the model output. The GPP and ER ratio (P/R) and net ecosystem production (NEP) (GPP – ER) were calculated.

Per land use type, 40 measurements were taken in total: 8 in the main flow path (the average of the two replicate probes in the main flow path × 4 streams, for each of the 2 days during the measurement period separately) and 32 in deposition zones (4 probes × 4 streams × 2 days).

### Macroinvertebrate community composition

In each stream four replicate macroinvertebrates samples were taken from the deposition zones using a Surber sampler (625 cm^2^; mesh size: 0.5 mm). The collected organisms were kept cold, sorted and within 48 hours identified to genus level. Total abundance and species richness were calculated.

### Statistics

Differences in substrate cover (log transformed data), water and sediment quality parameters and mean oxygen concentrations between land use types and between the main flow path and the deposition zones were tested separately using one-way analysis of variance (ANOVA), followed by a Tukey post hoc test (R-package stats). In those cases where the conditions of data normality (Shapiro–Wilk test) and homogeneity of variances (Levene’s test) were violated, differences between means were calculated using the non-parametric Kruskal–Wallis test, followed by a Mann-Whitney pairwise comparison test (R-package multcompView).

To test for differences in metabolism (GPP and ER rates) between land use types, linear effect models were used with land use type, within stream location and day as predictors (averaged replicate probes data was used) (R-packages lmertest and emmeans)^34,35^.

To relate the frequency of oxygen saturation recorded as low saturation (classes below 20%), medium saturation (from 21% to 50%), high saturation (from 51% to 100%) and supersaturation (above 100%) to the water and sediment quality parameters, log-transformed data from all replicate streams were included in a PCA, except for the correlated variables (correlation coefficient >0.8) (R-package Hmisc). PCA axis 1 and 2 were tested with a generalized linear model (GLM) performed in CANOCO for Windows version 5.12 (ter Braak & Smilauer, 2002).

To analyse the differences in macroinvertebrate community composition among streams, a nonmetric multidimensional scaling (NMDS) was performed with log transformed abundance data followed by an analysis of similarities (ANOSIM) to test differences between sites (R-package Vegan)^36^. The fitting of environmental variables to the ordination plot was performed with vegan and the significance was obtained with a 1000 permutations test. Tested parameters were water quality (conductivity, turbidity, NO_2_^−^, NO_3_^−^, NH4^+^and PO_4_^3−^), sediment characteristics (Chl*a*, OM, C, N, C/N, IP, OP, TP and phi), substrate cover (wood debris, sand, macrophytes, FPOM and CPOM) and oxygen (frequency of occurrence of oxygen saturation classes below 20%, from 21% to 50%, from 51% to 100% and above 100%).

Moreover, to identify specific taxonomic shifts associated with land use specific fine sediment inputs, an indicator species analysis was performed (R-package indicspecies)^37^.

## Results

### Water quality

pH and nitrate concentrations were similar in all streams. Turbidity, conductivity and nitrite, ammonium and total phosphorus concentrations differed significantly (p < 0.05) between land use types (Table 1). Turbidity was significantly (p < 0.05) higher in cropland streams than in all other streams. Conductivity was significantly (p < 0.05) higher in WWTP streams than in all other streams, except cropland streams. Conductivity was also significantly (p < 0.05) higher in IG and cropland streams than in forest and IG streams. Nitrite concentrations were highest in WWTP streams, but only significantly (p < 0.05) different from forest and EG streams. Ammonium concentrations in forest streams were significantly (p < 0.05) lower than in cropland streams. Total phosphorus concentrations were highest in WWTP streams, but only significantly (p < 0.05) different from EG and cropland streams.

**Table 1:** Physico-chemical characteristics of the selected streams.

		forest	EG	IG	cropland	WWTP
Water quality	pH	7.4 (0.3)^a^	7.5(0.02)^a^	7.9 (0.09)^a^	7.5 (0.5)^a^	7.5 (0.3)^a^
	Turbidity (NTU)	21.5 (16.5)^ab^	27.3 (12.9)^ab^	15.7 (4.7)^ab^	51.8 (20.4)^b^	10.1 (6.3)^a^
	EC (µS/cm)	184 (65)^a^	206 (43)^a^	295 (47)^b^	421 (100)^bc^	602 (76)^c^
	NO_2_^−^ (µM)	0.6 (0.7)^a^	0.4 (0.3)^a^	1.2 (0.7)^ab^	1.3 (0.5)^ab^	3.5 (3)^b^
	NO_3_^−^ (µM)	82.7 (70.1)^a^	86.9 (127.7)^a^	58.6 (39.4)^a^	205.6 (195.3)^a^	175.0 (34.4)^a^
	NH_4_^+^ (µM)	5.5 (6)^a^	10.1 (4.8)^ab^	18.3 (8.2)^ab^	154.9 (147.4)^b^	61.3 (74.6)^ab^
	PO_4_^3−^ (µM)	0.6 (0.5)^abc^	0.3 (0.1)^b^	1.4 (1.2)^c^	0.3 (0.1)^b^	2.5 (1.5)^cd^
Sediment quality	Grain size (Phi)	2.01 (0.31)^a^	2.63 (0.07)^a^	2.82 (0.15)^a^	2.94 (0.18)^a^	2.63 (0.28)^a^
	TC (mol/kg)	1.3 (0.3)^a^	3.5 (2.5)^a^	3.8 (3.2)^a^	6.5 (5.2)^a^	0.7 (0.1)^a^
	OM (%)	3.0 (0.6)^ab^	7.7 (5.2)^a^	7.9 (6.3)^a^	14.4 (11.5)^a^	1.4 (0.3)^b^
	TP (mmol/kg)	10.0 (1.9)^a^	37.1 (19.9)^a^	18.6 (10.8)^a^	146.2 (226.1)^a^	12.1 (4.4)^a^
	OP (mmol/kg)	3.1 (0.1)^a^	8.7 (8.5)^a^	5.3 (4.7)^a^	61.4 (102.7)^a^	4.1 (1.4)^a^
	IP (mmol/kg)	6.9(2)^a^	28.4 (12.3)^a^	13.3 (6.9)^a^	84.8 (123.6)^a^	8 (3.3)^a^
	TN (mol/kg)	0.06 (0.01)^a^	0.20 (0.14)^a^	0.20 (0.14)^a^	0.39 (0.32)^a^	0.05 (0.01)^a^
	Chla (mg/g)	6.7 (7.9)^a^	5.6 (2.1)^a^	41.1 (30)^b^	9.2 (9)^ab^	5.9 (5.2)^a^
	C/N	20.4 (2.6)^a^	17.8 (1.5)^ab^	17.9 (2.8)^ab^	16.6 (1.1)^bc^	13.9 (1.3)^c^
Substrate cover	Algae (%)	0 (0)^a^	0 (0)^a^	8.8 (17.5)^b^	0 (0)^a^	0 (0)^a^
	Macrophytes (%)	0 (0)^a^	52.1 (30.6)^b^	8.7 (8.0)^b^	27.4 (29.0)^b^	34.4 (28.9)^b^
	Wood debris (%)	6.6 (2.8)^b^	0 (0)^a^	0 (0)^a^	0 (0)^a^	0 (0)^a^
	Gravel (%)	0.3 (0.5)^a^	0 (0)^a^	0 (0)^a^	0 (0)^a^	3.8 (6.4)^a^
	Sand (%)	29.2 (21.2)^a^	0 (0)^b^	35.8 (15.3)^a^	0 (0)^b^	32.9 (31.3)^a^
	CPOM (%)	38.8 (23.1)^b^	0 (0)^a^	0 (0)^a^	0 (0)^a^	0 (0)^a^
	FPOM (%)	25.4 (11.9)^a^	46.7 (29.6)^a^	46.7 (19.7)^a^	72.9 (29.6)^a^	27.1 (34.2)^a^

Water quality parameters (pH, turbidity, conductivity (EC), nitrite (NO_2_^−^), nitrate (NO_3_^−^), ammonium (NH4^+^), phosphorus (PO_4_^3−^) and concentrations), sediment characteristics of the deposition zones (grain size, total carbon content (TC), organic matter content (OM %), total/organic/inorganic phosphorus content (TP/OP/IP), total nitrogen (N) content, chlorophyll–*a* (chla), content carbon/nitrogen ratio (C:N)) and substrate cover (in % estimated according to Hering *et al*. 2003) are given as means per land use type (n = 4 replicate streams). Standard deviations are given between brackets. Letters indicate a significant difference (p < 0.05) between land use types (Forest, EG – extensive grassland, IG – intensive grassland, cropland and WWTP – wastewater treatment plant) based on analyses of variance followed by multiple comparison test.

### Sediment characteristics

Chemical composition of the sediment in the deposition zones differed between land use types in organic matter content, chlorophyll-*a* concentration and C/N ratio (Table 1). Organic matter content was significantly (p < 0.05) lower in sediments from WWTP streams than in sediments from EG, IG and cropland streams. Sediment chlorophyll-*a* concentrations were significantly (p < 0.05) higher in intensive grassland streams than in all other streams, except from cropland streams. The sediment C/N ratio was significantly higher in forest streams than in cropland and WWTP streams.

### Substrate cover

Substrate cover differed between the streams depending on the land use type (Table 1). Algae were only found in IG streams, while macrophytes were found in all streams except from forest streams. Woody debris and CPOM were only found in forest streams. Gravel was found in low coverage percentages in forest and WWTP streams, while sand was found in forest, IG and WWTP streams. FPOM was found in relatively high (>25%) coverage percentages in all streams.

### Dissolved oxygen regime

**Figure 1:**
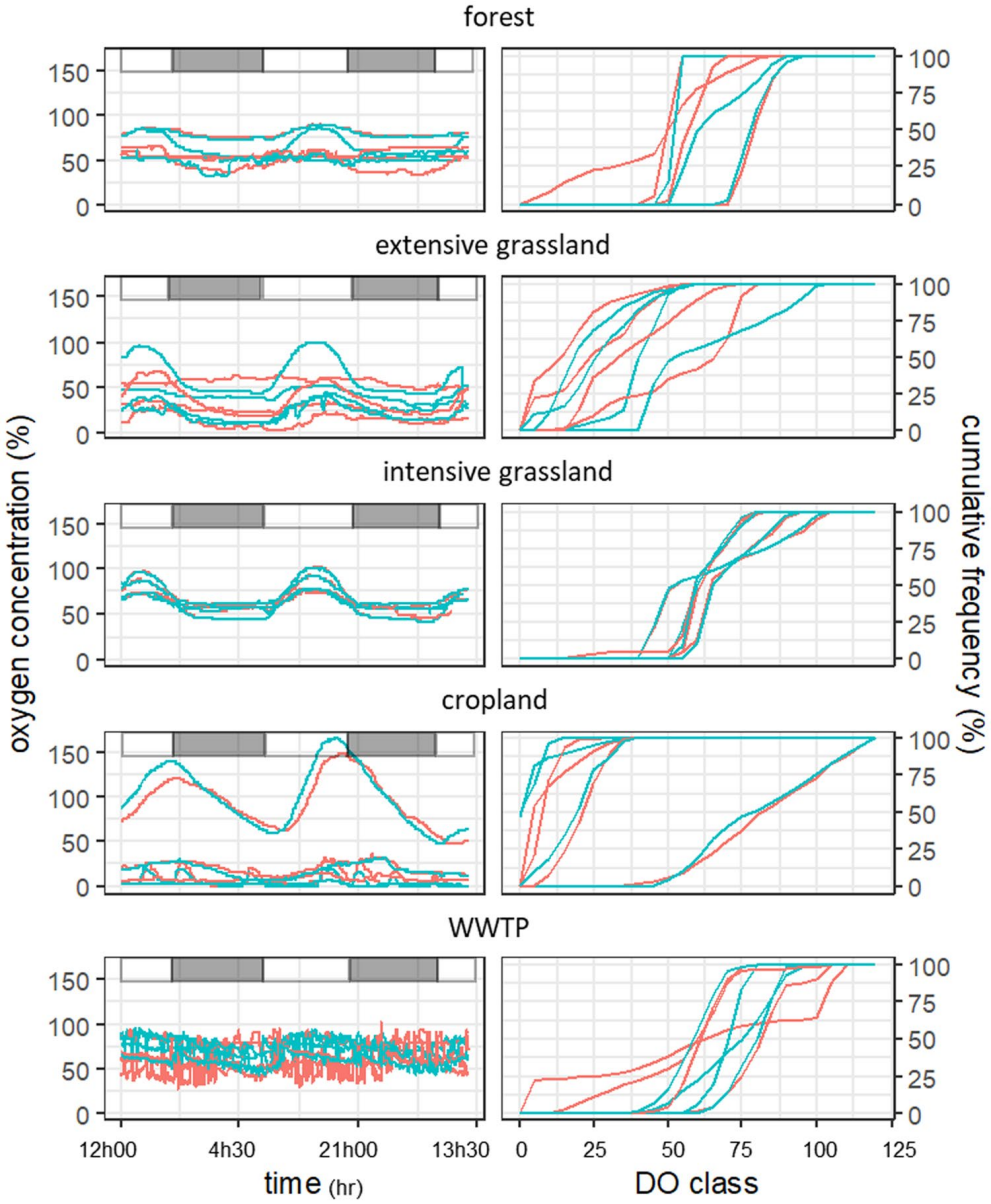
Dissolved oxygen concentrations (left panels; in % air saturation) and corresponding cumulative frequency distributions of the oxygen concentrations (right panels) measured during 48 hours in the main flow path of the stream (blue lines) and in the deposition zones (red lines) in 4 replicate streams per land use type. Light-dark periods are represented by white and grey rectangles respectively (left panels).

No significant differences (p > 0.05) between the main flow path and the deposition zones were observed. In contrast, large temporal variations in oxygen concentrations were observed, ranging from an average difference of 27 to 56% between the maximum and minimum DO concentrations in the forest streams and EG streams, respectively (Fig. 1 left panels). In a single replicate cropland streams a 117% difference between the maximum and minimum concentrations was observed. In all forest, EG, IG and two of the replicate cropland streams, the observed temporal variation coincided with the natural light-dark regime (Supplementary Fig. S3). In the other cropland streams and in all WWTP streams, fluctuations in dissolved oxygen concentrations showed an irregular pattern with a high frequency of changes, not related to natural variations in the daily light regime (Fig. 1).

The analysis of the cumulative frequency distributions shows the predominant dissolved oxygen saturation classes per land use type. More than half of the dissolved oxygen measurements in cropland streams were below 15% air saturation for both the main flow path and the deposition zones (Fig. 1, right panels). In contrast, in forest, intensive grassland and WWTP streams, more than half of the dissolved oxygen measurements were above 60%. In extensive grassland streams, dissolved oxygen measurements were mostly above 30%.

### Sediment oxygen demand and stream metabolism

**Figure 2:**
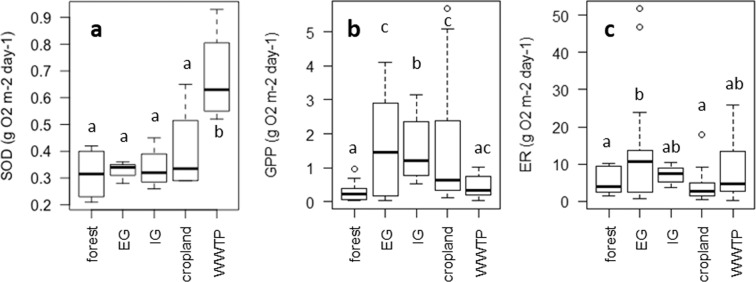
SOD (n = 4 replicate streams) (**A**) measured in sediment samples. GPP (**B**) and ER (**C**) based on BASEmetab model calculations in streams surrounded by different land use types. Because no significant differences per land use were found when the main flow path and the deposition zones were analysed separately, all GPP and ER measurements (n = 40 per land use type) were combined. Letters indicate a significant difference between land use types (p < 0.05).

The average sediment oxygen demand in WWTP streams (0.6 g O_2_/m^2^/day) was significantly (p < 0.05) higher than in forest, cropland and both types of grassland streams (around 0.3 g O_2_/m^2^/day) (Fig. 2a).

The metabolism measurement goodness of fit decreased from IG (mean R^2^ = 0.88, ± 0.11), EG (mean R^2^ = 0.72, ± 0.26) and forest (mean R^2^ = 0.67, ± 0.28) streams to cropland (mean R^2^ 0.59, ± 0.30) and WWTP (mean R^2^ = 0.32, ± 0.18) streams. Comparing land use types, the gross primary production was significantly (p < 0.05) lower in forest and WWTP streams compared to all other streams (Fig. 2b). Ecosystem respiration rates were significantly (p < 0.05) lower in forest and cropland streams than in EG and WWTP streams (Fig. 2c). NEP (average for all measurements including main flow and deposition zones) was therefore lower in EG (−11.48 ± 13.38) and WWTP (−8.03 ± 7.29) streams than in IG (−5.6 ± 1.28), forest (−5.07 ± 3.62) and cropland (−2.45 ± 4.34) streams. P/R mean was lower in forest (0.09 ± 0.09) streams than in WWTP (0.13 ± 0.12), IG (0.2 ± 0.08), cropland (0.58 ± 0.39) and EG (0.69 ± 2.31) streams, although no significant differences were found for NEP. There was no significant difference in metabolic rates (GPP, ER, P/R and NEP) between the main flow path and the deposition zones for the studied streams (Supplementary Table S2).

### Relationship between environmental conditions and oxygen concentrations

**Figure 3:**
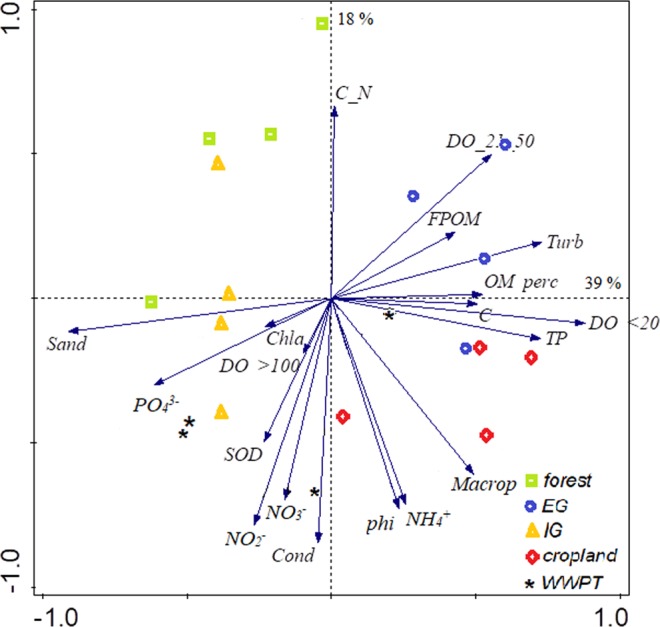
Principal Component Analysis (PCA) biplot for ordination of dissolved oxygen concentrations (based on frequency distributions of the concentrations in Fig. 1), sediment characteristics and water quality in 20 lowland streams from five different land use types (Forest, EG- extensive grassland, IG - intensive grassland, Crop- cropland and WWTP).

In Fig. 3, streams from different land use types were ordinated based on the frequency of oxygen saturation classes and the measured environmental variables. Oxygen saturation classes were categorized as low saturation (below 20% (DO < 20), medium saturation (from 21% to 50% (DO 21-50)), high saturation (from 51% to 100% (DO 51-100)) and supersaturation (above 100% (DO > 100) (data from Fig. 1, right panels). The correlated (correlation coefficient > 0.8) variables DO 51-100, OP, IP and N were removed from the PCA analyses. The first two axis of the PCA explained 56.8% of the variation. The first axis clearly separated all cropland streams and extensive grassland streams based on low oxygen saturation (DO < 20), high turbidity, substrate cover of fine particulate organic matter, a high sediment organic matter content and higher sediment nutrient (TP, C) concentrations, since these environmental variables significantly (p < 0.005) correlated with axis 1. The second axis separated forest streams from WWTP streams based on C/N ratio and dissolved nutrient content (NO_2_^−^, NH_4_^+^, NO_3_^−^), conductivity and grain size (phi), significantly (p < 0.005) correlated with axis 2.

### Macroinvertebrate community composition

**Figure 4:**
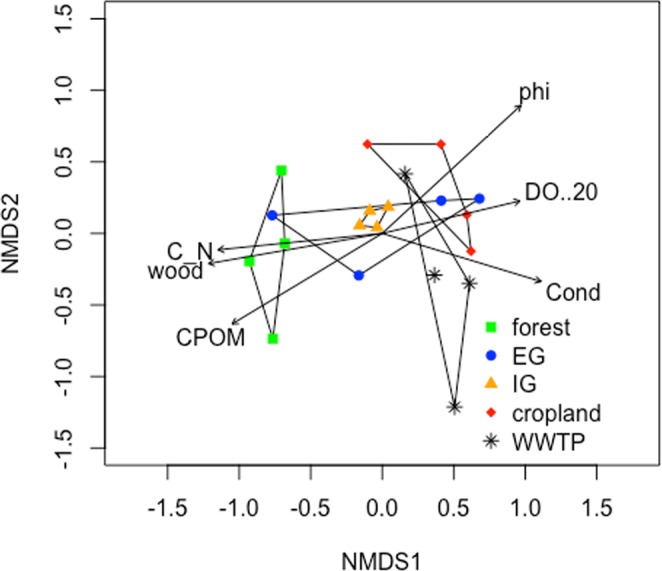
Non-Metric Multidimensional Scaling (NMDS) ordination of macroinvertebrate community (stress = 0.19; two dimensions; non-metric fit R2 = 0.96; linear fit R2 = 0.8). Contour polygons group the assemblages per land uses type (package Vegan; metaMDS; bray distance; monoMDS). The arrows correspond to the significant (p < 0.05) environmental variables measured in the stream deposition zones (data in Table 1).

All streams were characterized by a typical lowland stream macroinvertebrate community (Supplementary Table S3). The average abundance varied from 224 individuals per sample in the streams surrounded by extensive grasslands to 743 in the WWTP streams. No large differences in number of taxa (16–19 taxa) were observed between the streams. However, there was a dominance of *Chironomus* sp., Oligochaeta and Gastropoda in cropland and WWTP streams, while Plecoptera and most Trichoptera only occurred in forest and extensive grassland streams (Supplementary Table S3). The non-metric multidimensional scaling (NMDS diagram) clearly separated the macroinvertebrate communities based on land use types (Fig. 4, ANOSIM: r = 0.2; p = 0.03). Only the communities from extensive grassland streams showed large variations between replicates. Forest streams were located on the left side of the ordination graph and were related to a high oxygen saturation, a high C/N ratio in the sediment and woody debris and CPOM substrate cover. On the opposite side, the macroinvertebrate community composition in cropland and WWTP streams were related to a low oxygen saturation, a high conductivity and a small grain size (high phi). Considering specific taxa contributing to the observed site grouping, two Trichoptera taxa were indicator species for forest streams, Ostracoda for intensive grassland streams, while *Valvata* sp., *Stylaria lacustris, Chironomus* sp. and *Helobdella stagnalis* were indicators for WWTP streams (Supplementary Table S4).

## Discussion

We showed that differences in surrounding land use determine physico-chemical stream characteristics, ecosystem functioning and macroinvertebrate community composition in lowland streams. By measuring these interrelated components of ecosystem structure and function simultaneously, the present study also contributed to unravelling the underlying mechanisms, as discussed below.

### Physico-chemical stream characteristics

Differences in substrate cover (e.g. FPOM dominance), sediment organic matter content (e.g. higher nutrient concentrations) and some water quality parameters (e.g. turbidity and dissolved nutrients concentrations) were observed in streams in which the catchment is dominated by human impacts compared to natural lowland streams surrounded by forests. These differences are most likely related to the human activities in the catchment, such as the use of fertilizers, ploughing and the presence of livestock in the agricultural fields^38^, or the treatment of wastewaters^39^. Substrate cover was affected in all human impacted streams (grassland, cropland and WWTP), particularly expressed by an increase in fine particulate organic matter and/or an increase in the presence of aquatic macrophytes when compared to the forest streams. Although not directly reflected by the nutrient concentrations in this study, it could indeed be expected that especially in streams surrounded by open fertilized agricultural grasslands both light and nutrients are not limiting, resulting in the enhanced growth of macrophytes^40^. Also an increase in sediment organic matter content and sediment chlorophyll concentrations was found in streams surrounded by agricultural fields, pointing to the input of nutrient rich organic particles from these fields. Water quality was most affected in the WWTP streams, as a direct result from the input of effluent from the wastewater treatment plants. Similar impacts of land use have been shown previously by e.g. Wood and Armitage^41^; Walsh *et al*.^39^.

### Ecosystem functioning

The observed differences in physico-chemical stream characteristics coincided with clear differences in stream metabolism rates and oxygen regimes. The low productivity/respiration ratios (<1 in this study) indicate that all studied lowland streams, regardless of land use type, depended on energy input from the catchment^42^, hence being predominantly fuelled by allochthonous organic matter and nutrient inputs from the adjacent land^43^. To quantify the effects on stream metabolism we analyzed oxygen regimes, but the BASEmetab model could not deal very well with the artificial daily variation in oxygen regime as observed in the WWTP streams, affecting the reliability of the stream metabolism results.

As indicated by the present principal component analysis, the terrestrial input of particles and nutrients subsequently resulted in differences in oxygen concentrations, varying with land use type. Moreover, besides the impact on the average dissolved oxygen concentrations, also strong differences in diel variations in oxygen concentrations were observed in streams surrounded by different land use types. In streams surrounded by croplands, the long periods of low oxygen concentrations are possibly linked to the influx of organic particles from the adjacent field, as indicated by the high turbidity and a high percentage of FPOM substrate cover and a low turbulence, characteristic of low gradient streams. The high FPOM substrate cover and water turbidity hamper the development of primary producers, as previously observed by Jones *et al*.^44^ and therewith diminish the oxygen concentration in the water column. In contrast, in streams surrounded by grasslands, the relatively strong daily oxygen fluctuations coinciding with the light-dark cycle were most likely caused by the macrophytes growing in the nutrient rich deposition zones with a high light availability. As a result, the grassland streams also showed the highest ecosystem production rates. These findings corroborate previous studies by e.g. Finlay^45^ and Bernot^46^, who reported that streams located in open fields, such as agricultural grasslands, showed higher primary production than forest streams. In contrast, fluctuations in oxygen concentrations in the WWTP streams were not related to the natural daily variations in light and primary production, but instead showed an irregular, fast fluctuating pattern, which was most likely caused by the frequent and fluctuating discharge of effluent from the nearby sewage treatment plants. Treatment plants can cause typical increased hydrologic flashiness of the effluent-receiving streams^39,47,48^, causing disruption of the natural dissolved oxygen concentrations patterns in the receiving waters. Although the average oxygen concentration in the water of the WWTP streams was quite high, the oxygen availability in the sediment is expected to be low, as a result of the presently observed high sediment oxygen demands and high respiration rates in the WWTP streams. This high benthic metabolic activity observed in the studied WWTP streams coincided with the continuous input of nitrate, phosphorus and oxygen into the water column, likely stimulating the microbial community growing on the top sediment layer of the wastewater impacted streams^39,49–51^. The observed effects on oxygen regimes in streams surrounded by human impacted land use types are in agreement with Young, Matthaei, and Townsend^52^, who suggested that impacted streams deviate from natural forest streams in terms of gross primary production and community respiration and with Battin *et al*.^15^ and Lear *et al*.^14^, who argued that fundamental ecosystem processes were driven by metabolic activities from the microbial community growing on top of the sediment.

### Macroinvertebrate community composition

Ultimately, the differences in physico-chemical stream characteristics and oxygen regimes in the streams flowing through different land use types were also reflected by differences in macroinvertebrate community structure. The specific oxygen demands of the individual species^53^ appeared to be an important ecological driver behind the observed patterns, in line with Berger *et al*.^54^, who observed that most macroinvertebrate taxa responded sensitively to the prevailing oxygen concentrations. While the long periods of low dissolved oxygen concentrations in the water column may explain the difference in community composition in cropland streams compared to forest streams, in WWTP streams it was mainly the expected low oxygen concentrations in the sediment that might have affected community composition^55^. In cropland and WWTP streams, the low oxygen availability and fine sediment limited the occurrence of oxygen sensitive EPT (Ephemeroptera, Plecoptera and Trichoptera) species^56,57^, while taxa that can withstand frequent low dissolved oxygen concentrations, such as *Chironomus* sp., worms and gastropods, dominated the macroinvertebrate communities^12,54,58,59^. In the grassland streams, the oxygen concentration regimes did not seem to be a limiting factor for the macroinvertebrates, since long periods of high oxygen concentrations were recorded in these streams at least during daytime. Yet, according to the NMDS analyses, the lower oxygen concentrations during the night in the grassland streams may have caused these communities to take a position in between the forest streams and the heavily impacted cropland and WWTP streams macroinvertebrate communities. We acknowledge however, that effects on macroinvertebrate communities arising from other land use related stress, such as habitat homogeneity^4^, hydrologic disturbance^2^ and contaminant loads^60^ might also occur.

### Synthesis and conclusions

The present study provided new insights in the relationship between the surrounding land use type and stream metabolism and oxygen regime in lowland streams. Streams receiving forest inputs (leaves and woody debris) sustained a relatively stable high oxygen regime and a diverse macroinvertebrate community. In grassland streams, the nutrient-rich sediments promoted the activity of primary producers, resulting in highly fluctuating oxygen regimes with effects on macroinvertebrates community composition. Silt driven cropland streams were characterized by low oxygen concentrations and high abundances of taxa insensitive to low dissolved oxygen concentrations. Streams receiving WWTP effluents showed a high heterotrophic microbial community activity, high sediment oxygen demands and were inhabited by macroinvertebrate communities indicative of deteriorated conditions.

We have thus confirmed the hypothesis that differences in catchment land use type determine physicochemical stream characteristics, ecosystem functioning and macroinvertebrate community structure in lowland streams. Therefore, we argue that land use specific impacts on lowland streams were exerted via fine sediment accumulation in deposition zones, affecting oxygen regimes, sediment oxygen demand and stream metabolism, ultimately changing macroinvertebrate community composition.

This study supports therefore the importance of including the catchment scale in ecological stream quality assessments, combining structural and functional endpoints.

## Supplementary information

Sup. material

## Data Availability

The authors declare that the data supporting the findings of this study are available within the paper and its Supplementary Information files.
