# Supplementary material for: Land use affects lowland stream ecosystems through dissolved oxygen regimes
Source: Sci Rep. 2019 Dec 23;9:19685. doi: 10.1038/s41598-019-56046-1 (PMC6927968; doi:10.1038/s41598-019-56046-1)
Supplement: Supplementary file 1 — Sup. material [file 41598_2019_56046_MOESM1_ESM.pdf]

Land use affects lowland stream ecosystems through dissolved oxygen regimes

Paula C. dos Reis Oliveira
Harm G. van der Geest
Michiel H. S. Kraak
Piet F. M. Verdonschot

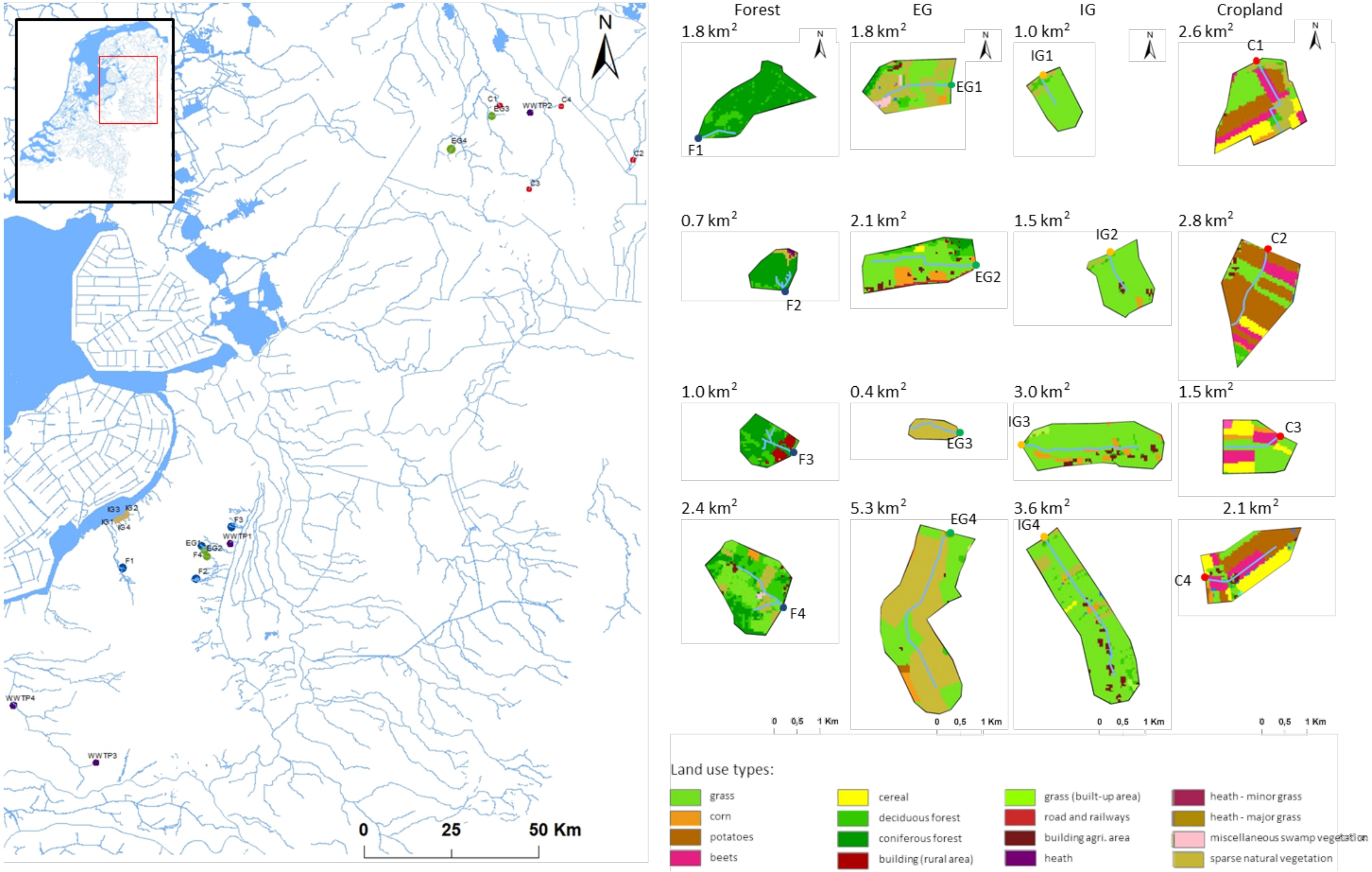

Figure S1: Sampling sites of twenty streams (Forest: F1, F2, F3 and F4; extensive grassland: EG1, EG2, EG3 and EG4; intensive grassland: IG1, IG2, IG3 and IG4; cropland: C1, C2, C3 and C4; WWTP: WWTP1, WWTP2, WWTP3 and WWTP4) within the network in water bodies in the Netherlands. Sub-catchment boundary was delimitated by elevation data or man-made structures in the landscape (e.g. drainage ditches) in Forest, EG, IG and cropland land use types based on the topographic map of the Kadaster [1:25.000 scale] at RD new projection. Image created with Esri, ArcGIS Desktop 10.6 , 2019.

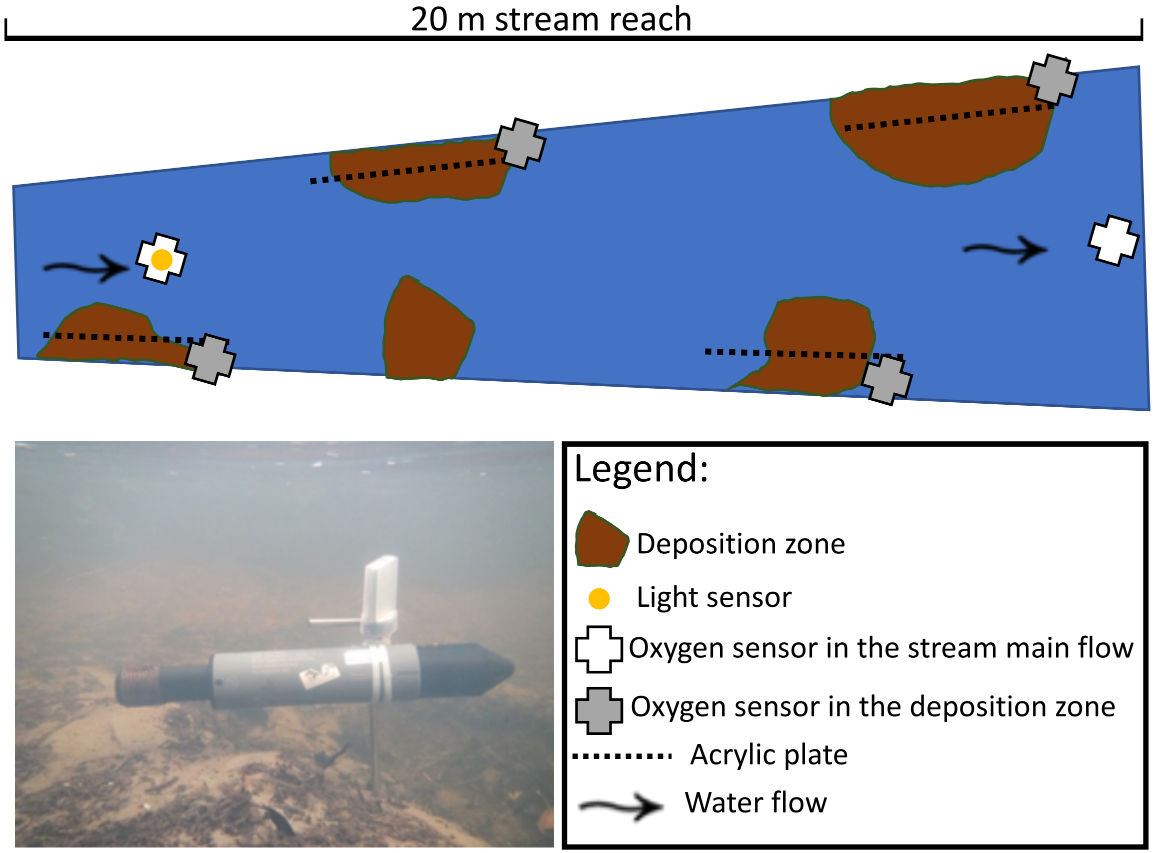

Figure S2: Installation of oxygen and light sensors in each studied stream.

Table S1: Mean water temperature (℃), depth (m); width (m), velocity (m/s) and catchment area (n = 4 replicate stream), GPS coordinates (decimal degrees) and the percentage occurrence of each land use type present in the catchment, per land use type (Forest, EG – extensive grassland, IG – intensive grassland, cropland and WWTP – wastewater treatment plant).

	forest	EG	IG	cropland	WWTP
Water temperature (℃)	12.6 (1.0)	12.4 (0.4)	12.8 (0.1)	14.7 (0.5)	15.9 (0.8)
Depth (m)	0.13 (±0.08)[a]	0.27 (±0.15)[a]	0.22 (±0.02)[a]	0.35 (±0.1)[a]	0.26 (±0.07)[a]
Width (m)	2.0 (±0.3) [a]	2.2 (±1.2) [a]	2.4 (±0.8) [a]	2.9 (±1.2) [a]	2.8 (±0.7)[a]
Velocity (m/s)	0.099 (±0.02)[ab]	0.069 (±0.08)[a]	0.118 (±0.03)[ab]	0.022 (±0.01)[a]	0.192 (±0.05)[b]
Discharge (m3/s)	0.004 (±0.002)[a]	0.003 (±0.002)[a]	0.010 (±0.006)[ab]	0.004 (±0.004)[a]	0.022 (±0.012)[b]
Total catchment area (km2)	1.5 (± 0.8)	2.4 (± 2.0)	2.3 (± 1.2)	2.2 (±0.5)	n.a.
GPS coordinates (DD)					
replicate 1	52.305007, 5.726070	52.332772, 5.937832	51.995054, 5.653381	53.026983, 6.698200	52.343681, 6.001478
replicate 2	52.288031, 5.915494	52.322866, 5.944421	52.3874072,5.730138	52.9422384,7.0401342	53.014682, 6.774243
replicate 3	52.3698969,6.0065093	52.9364721,6.6275572	52.389585, 5.733787	52.8947691,6.7682192	51.995914, 5.653480
replicate 4	52.340030, 5.929707	52.9592017,6.571805	52.382086, 5.711314	53.025835, 6.860144	52.089662, 5.446765
Land use type					
forest (%)	92 (±9.3)	1.8 (±1.9)	0 (±0)	2 (±3)	n.a.
EG (%)	9.4 (±13.3)	88.1 (±8.9)	5 (±5.1)	0 (±0)	n.a.
IG (%)	0 (±0.4)	9.3 (±9.7)	88.9 (±2.9)	25.4 (±5.7)	n.a.
cropland (%)	1.6 (±0.8)	0.8 (±1.6)	2.8 (± 2.9)	72.6 (±3.3)	n.a.
urban (%)	0 (±3.7)	0 (±0)	3.2 (±2.2)	0 (±0)	n.a.

Table S2: Mean gross primary production (GPP) respiration (ER), the productivity and respiration ratio (P/R) and net ecosystem production (NEP) per land use type for main path flow (n = 8) and deposition zone (n = 32).

	forest		EG		IG		cropland		WWTP	
	main flow path	deposition zone	main flow path	deposition zone	main flow path	deposition zone	main flow path	deposition zone	main flow path	deposition zone
GPP (g m^{-2} d^{-1})	0.45 (0.40)	0.23 (0.22)	1.68 (0.99)	1.49 (1.52)	1.66 (1.06)	1.79 (1.40)	1.88 (2.58)	1.36 (1.63)	0.43 (0.33)	0.44 (0.32)
ER (g m^{-2} d^{-1})	5.43 (3.12)	5.33 (3.73)	11.43 (9.60)	13.39 (15.22)	8.08 (2.04)	6.89 (2.06)	3.78 (2.21)	3.94 (4.43)	3.20(0.67)	9.79 (7.90)
P/R	0.11 (0.12)	0.08 (0.09)	0.18 (0.13)	0.15 (0.12)	0.19 (0.08)	0.25 (0.18)	0.47 (0.43)	0.54 (0.39)	0.15 (0.13)	0.08 (0.09)
NEP (g m^{-2} d^{-1})	-4.98 (3.32)	-5.1 (3.81)	-9.75 (8.90)	-11.9 (14.49)	-6.42 (1.13)	-5.10 (1.68)	-1.9 (1.89)	-2.77 (0.87)	-2.77 (0.87)	-9.35 (7.61)

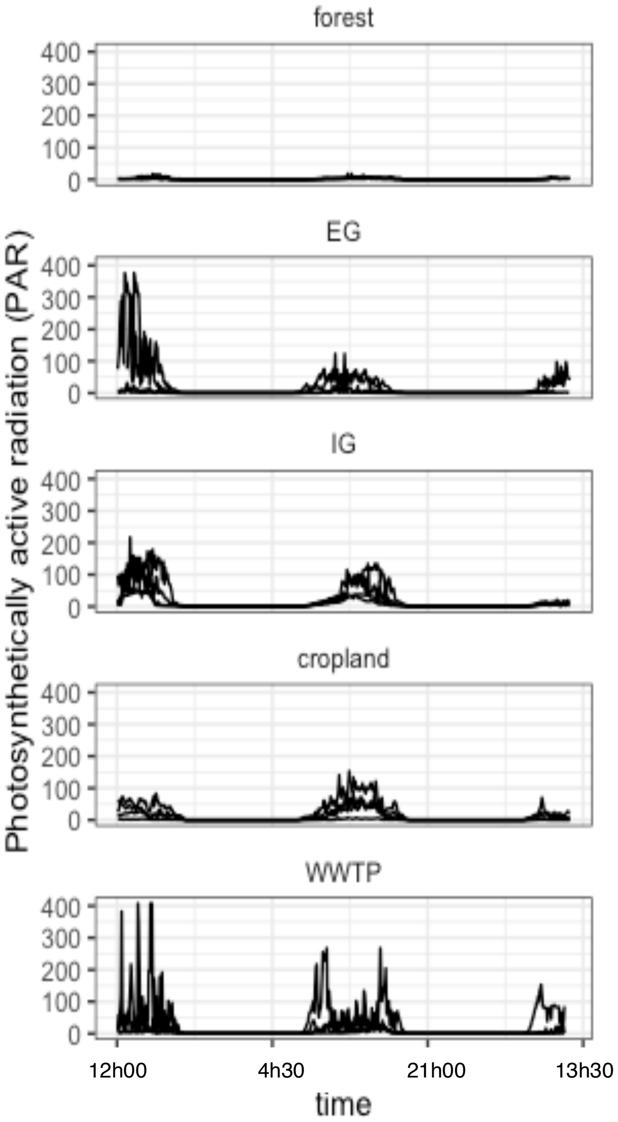

Figure S3: Light in photosynthetically active radiation (PAR) measured during 48 hours in the main flow path of the stream in 4 replicate streams per land use type.

Table S3: Mean abundance and standard deviation (sd) (n = 4) of macroinvertebrate taxa per sample (625 cm^2), per land use (Forest, EG – extensive grassland, IG – intensive grassland, cropland and WWTP – wastewater treatment plant).

		forest		EG		IG		cropland		WWTP	
		mean	sd	mean	sd	mean	sd	mean	sd	mean	sd
Asselidae	*Asellus aquaticus*	5.9	10.9	9.3	9.4	50.3	62.8	14.5	12.7	32.4	38.8
	Proasellus sp.	0.1	0.1	0.3	0.6	2.4	3.5	0.2	0.4	14.8	28.8
Bivalvea	*Pisidium* sp.	5.6	1.8	34.3	28.6	10.5	11.0	23.2	17.9	0.1	0.3
	Sphaerium sp.	0.0	0.0	1.8	3.1	0.0	0.0	11.8	16.2	0.8	1.3
Chironomidae	*Apsectrotanypus trifascipennis*	4.4	7.4	1.0	1.7	10.8	8.3	0.0	0.0	0.0	0.0
	Chironomus sp.	0.8	0.7	1.6	2.8	0.6	0.7	7.7	8.6	33.3	48.5
	Cladopelma sp.	0.0	0.0	0.0	0.0	0.0	0.0	0.0	0.0	0.1	0.1
	Cladotanytarsus sp.	0.1	0.1	0.0	0.0	4.4	8.4	0.0	0.0	0.0	0.0
	Clinotanypus nervosus	0.0	0.0	0.1	0.3	2.4	3.1	1.1	1.9	0.8	1.3
	Conchapelopia sp.	0.6	0.5	0.1	0.1	0.6	0.8	0.1	0.1	0.1	0.1
	Corynoneura sp.	0.3	0.2	0.0	0.0	0.1	0.1	0.0	0.0	0.0	0.0
	Cricotopus sp.	0.0	0.0	0.0	0.0	0.0	0.0	0.0	0.0	0.3	0.3
	Cryptochironomus sp.	0.1	0.3	0.0	0.0	1.4	1.8	0.1	0.3	0.4	0.5
	Dicrotendipes sp.	0.0	0.0	0.0	0.0	0.0	0.0	0.2	0.4	0.0	0.0
	Endochironomus sp.	0.0	0.0	0.1	0.1	0.1	0.3	0.6	0.7	1.6	3.3
	Epoicocladius sp.	0.4	0.9	0.3	0.5	0.0	0.0	0.0	0.0	0.0	0.0
	Eukiefferiella sp.	0.1	0.1	0.0	0.0	0.0	0.0	0.0	0.0	0.0	0.0
	Glyptotendipes sp.	0.0	0.0	0.0	0.0	0.0	0.0	0.1	0.3	0.0	0.0
	Heterotrissocladius sp.	0.2	0.4	0.0	0.0	0.0	0.0	0.3	0.5	0.0	0.0
	Macropelopia sp.	2.1	4.0	0.2	0.4	0.8	0.7	0.4	0.8	0.0	0.0
	Micropsectra sp.	172.8	340.0	0.8	1.2	16.5	12.7	0.0	0.0	0.0	0.0
	Microtendipes sp.	0.0	0.0	0.0	0.0	0.1	0.3	0.1	0.1	5.4	9.9

	Parachironomus sp.	0.0	0.0	0.1	0.1	0.1	0.1	0.1	0.3	0.4	0.8
	Paratendipes sp.	8.4	15.8	0.1	0.1	3.2	1.9	0.1	0.1	0.0	0.0
	Phaenospectra sp.	3.5	3.8	0.1	0.1	0.0	0.0	0.3	0.6	0.8	1.3
	Polypedilum sp.	5.9	7.8	0.6	0.7	0.4	0.6	0.4	0.8	0.1	0.1
	Procladius sp.	0.9	1.3	0.8	0.5	7.2	2.9	6.0	11.0	0.0	0.0
	Prodiamesa rufovittata	0.1	0.3	0.0	0.0	0.0	0.0	0.0	0.0	0.0	0.0
	Prodiamesa olivacea	4.1	2.8	0.4	0.6	1.8	2.2	0.0	0.0	0.8	1.1
	Psectrotanypus varius	0.0	0.0	9.7	18.7	0.4	0.3	11.5	13.8	0.3	0.5
	Rheotanytarsus sp.	0.2	0.4	0.3	0.6	0.1	0.3	0.0	0.0	0.1	0.1
	Schineriella schineri	0.3	0.4	0.0	0.0	0.0	0.0	0.0	0.0	0.0	0.0
	Stempelinella sp.	0.3	0.5	0.0	0.0	0.0	0.0	0.0	0.0	0.0	0.0
	Stichochironomus sp.	5.8	6.7	0.0	0.0	4.1	6.1	0.3	0.6	0.0	0.0
	Tanytarsus sp.	0.7	1.2	0.1	0.3	0.2	0.2	0.3	0.6	0.2	0.2
	Xenopelopia sp.	0.0	0.0	0.0	0.0	0.0	0.0	0.6	1.3	0.0	0.0
	Zavrelimyia sp.	1.1	1.2	0.0	0.0	0.1	0.3	0.0	0.0	0.0	0.0
	Agabus sp.	0.4	0.3	0.0	0.0	0.1	0.1	0.1	0.1	0.0	0.0
	Brychius sp.	0.0	0.0	0.0	0.0	0.1	0.1	0.0	0.0	0.0	0.0
	Colymbetes sp.	0.0	0.0	0.1	0.1	0.0	0.0	0.1	0.1	0.0	0.0
	Cyphon sp.	0.0	0.0	0.1	0.3	0.0	0.0	0.0	0.0	0.0	0.0
	Donaciinae	0.0	0.0	0.4	0.8	0.0	0.0	0.0	0.0	0.0	0.0
	Dryops sp.	0.0	0.0	0.0	0.0	0.1	0.1	0.0	0.0	0.1	0.3
	Dysticus sp.	0.4	0.9	0.0	0.0	0.0	0.0	0.0	0.0	0.0	0.0
	Elmidae	0.0	0.0	0.0	0.0	0.0	0.0	0.0	0.0	0.1	0.1
	Graptodytes sp.	0.0	0.0	0.0	0.0	0.1	0.1	0.0	0.0	0.0	0.0
	Gyrinus sp.	0.0	0.0	0.0	0.0	0.0	0.0	0.3	0.6	0.2	0.2
	Haliplus sp.	0.0	0.0	1.9	3.8	0.8	0.7	0.2	0.2	0.3	0.2
Coleoptera	*Hydroporus* sp.	0.2	0.4	0.7	0.8	0.3	0.3	0.5	0.8	0.0	0.0
	Hygrobia hermanni	0.0	0.0	0.1	0.1	0.0	0.0	0.0	0.0	0.0	0.0

	Hygrotus sp.	0.0	0.0	0.3	0.4	0.2	0.4	0.0	0.0	0.1	0.1
	Hyphydrus ovatus	0.0	0.0	0.8	1.3	0.1	0.1	0.0	0.0	0.0	0.0
	Ilybius sp.	0.2	0.4	0.0	0.0	0.0	0.0	0.0	0.0	0.0	0.0
	Platambus maculatus	1.2	1.5	0.0	0.0	0.1	0.1	0.0	0.0	0.0	0.0
	Porhydrus sp.	0.0	0.0	0.2	0.4	0.0	0.0	0.0	0.0	0.0	0.0
(other) Diptera	Ceratopogonidae	0.3	0.3	0.3	0.5	1.7	1.1	0.4	0.7	0.6	1.0
	Chaoboridae	0.0	0.0	0.3	0.6	0.0	0.0	0.0	0.0	0.0	0.0
	Culicidae	0.1	0.1	0.0	0.0	0.0	0.0	0.1	0.1	0.0	0.0
	Dicranota	0.1	0.1	0.0	0.0	0.0	0.0	0.0	0.0	0.0	0.0
	Dixella sp.	0.0	0.0	0.1	0.1	0.0	0.0	0.1	0.1	0.0	0.0
	Elodes sp.	0.2	0.4	0.6	1.3	0.0	0.0	0.0	0.0	0.0	0.0
	Eloeophila sp.	1.6	1.9	0.1	0.1	0.0	0.0	0.0	0.0	0.1	0.1
	Empididae	0.1	0.1	0.0	0.0	0.0	0.0	0.0	0.0	0.1	0.1
	Ephydridae	0.0	0.0	0.0	0.0	0.0	0.0	0.0	0.0	0.6	1.0
	Erioptera sp.	0.0	0.0	0.0	0.0	0.0	0.0	0.3	0.5	0.0	0.0
	Euphylidoea sp.	0.1	0.1	3.6	7.3	0.0	0.0	0.0	0.0	0.0	0.0
	Hexatoma sp.	0.1	0.1	0.0	0.0	0.0	0.0	0.0	0.0	0.0	0.0
	Neolimnomyia sp.	0.3	0.4	0.0	0.0	0.0	0.0	0.0	0.0	0.0	0.0
	Pilaria sp.	0.8	0.9	0.0	0.0	0.0	0.0	0.0	0.0	0.0	0.0
	Ptychopteridae	0.1	0.1	0.0	0.0	0.0	0.0	0.0	0.0	0.0	0.0
	Simuliidae	0.0	0.0	0.0	0.0	0.0	0.0	0.0	0.0	0.1	0.1
	Stratiomyidae	0.0	0.0	0.1	0.1	0.0	0.0	0.0	0.0	0.4	0.7
	Tabanidae	1.0	1.2	0.1	0.1	0.2	0.4	0.0	0.0	0.1	0.1
	Tipulidae	0.0	0.0	0.0	0.0	0.3	0.4	0.4	0.8	0.3	0.4
Ephemeroptera	*Baetis* sp.	0.0	0.0	0.0	0.0	0.1	0.1	0.0	0.0	0.0	0.0
	Caenis sp.	0.0	0.0	0.1	0.1	0.1	0.1	1.2	2.4	0.1	0.1
	Cloeon dipterum	0.5	1.0	10.6	13.7	1.3	1.1	66.8	104.6	0.3	0.2
	Leptophlebia sp.	11.6	23.1	0.0	0.0	0.0	0.0	0.0	0.0	0.0	0.0

	Procloeon bifidum	0.0	0.0	0.1	0.1	0.0	0.0	0.0	0.0	0.0	0.0
	Ephemera danica	5.3	9.2	2.5	5.0	0.0	0.0	0.0	0.0	0.0	0.0
Gammaridae	*Crangonix* sp.	0.0	0.0	0.9	1.4	0.0	0.0	0.6	1.3	10.5	21.0
	Gammarus roeselli	1.7	3.4	0.0	0.0	0.0	0.0	0.0	0.0	0.0	0.0
	Gammarus sp.	27.9	35.2	76.7	145.6	21.7	17.7	1.6	3.3	31.8	52.1
Gastropoda	*Anisus* sp.	0.3	0.5	0.9	1.1	3.6	3.3	5.9	6.9	1.8	3.5
	Bathyomphalus contortus	0.0	0.0	0.3	0.5	1.7	1.1	1.5	2.5	0.1	0.3
	Bithynia sp.	0.0	0.0	5.1	7.2	3.8	7.1	12.3	23.5	2.3	2.2
	Galba truncatula	0.0	0.0	0.1	0.1	0.0	0.0	0.0	0.0	0.1	0.3
	Gyraulus sp.	0.0	0.0	0.2	0.2	0.3	0.2	2.4	4.8	8.6	15.0
	Hippeutis complanata	0.0	0.0	0.2	0.4	0.0	0.0	0.7	0.6	0.1	0.1
	Lymnaea stagnalis	0.0	0.0	0.0	0.0	0.0	0.0	0.3	0.5	0.4	0.8
	Physa sp.	0.0	0.0	0.9	1.6	1.8	2.3	0.4	0.3	1.4	1.6
	Physella acuta	0.0	0.0	0.0	0.0	0.0	0.0	0.0	0.0	0.3	0.5
	Planorbarius corneus	0.0	0.0	1.4	1.1	0.1	0.1	1.8	2.0	0.5	0.8
	Planorbis sp.	0.0	0.0	1.5	3.0	0.9	1.6	0.2	0.4	0.4	0.8
	Potamopyrgus antipodarum	0.1	0.1	0.0	0.0	1.3	2.1	0.0	0.0	224.2	443.1
	Radix sp.	0.0	0.0	0.4	0.5	1.5	2.7	0.3	0.3	0.6	1.0
	Stagnicola sp.	0.0	0.0	0.6	1.3	0.1	0.1	0.1	0.1	0.3	0.3
	Valvata sp.	0.0	0.0	3.3	4.4	9.3	7.5	18.4	31.1	12.5	16.6
Hemiptera	*Cymatia* sp.	0.0	0.0	0.0	0.0	0.0	0.0	0.1	0.1	0.0	0.0
	Notonecta sp.	0.0	0.0	0.5	0.7	0.0	0.0	0.1	0.1	0.0	0.0
	Plea minutissima	0.0	0.0	0.1	0.1	0.0	0.0	0.2	0.4	0.0	0.0
	Sigara sp.	0.0	0.0	0.3	0.3	4.8	8.6	0.4	0.4	0.4	0.9
Hydracarina	Hydracarina	4.8	8.3	2.1	1.5	13.7	4.3	0.6	0.6	0.1	0.1
Hirudinea	*Alboglossiphonia* sp.	0.1	0.1	0.1	0.1	0.1	0.1	0.3	0.5	0.8	1.5

	Erpobdella sp.	0.8	0.9	0.3	0.2	1.4	1.3	0.6	0.6	8.5	7.1
	Glossiphonia sp.	0.8	1.2	0.4	0.5	0.9	1.5	0.3	0.4	1.6	1.9
	Helobdella stagnalis	0.0	0.0	0.0	0.0	0.8	1.3	0.8	1.3	28.5	55.7
	Theromyzon sp.	0.1	0.3	0.0	0.0	0.0	0.0	0.0	0.0	0.1	0.1
Lepdoptera	*Eleophila nymphaeata*	0.0	0.0	0.0	0.0	0.0	0.0	0.1	0.1	0.0	0.0
Megaloptera	*Sialis fuliginosa*	1.8	3.6	0.0	0.0	0.0	0.0	0.0	0.0	0.0	0.0
	Sialis lutaria	0.4	0.5	3.5	2.3	5.4	3.8	5.2	8.7	0.0	0.0
Odonata	*Aeshna* sp.	0.0	0.0	0.0	0.0	0.0	0.0	0.0	0.0	0.0	0.0
	Calopterix sp.	0.0	0.0	0.0	0.0	0.0	0.0	0.0	0.0	0.4	0.9
	Coenagrionidae	0.0	0.0	0.6	1.1	0.1	0.1	1.3	2.3	0.3	0.4
	Hesperocorixa sp.	0.0	0.0	0.1	0.1	0.0	0.0	0.0	0.0	0.0	0.0
	Libellula depressa	0.0	0.0	0.0	0.0	0.0	0.0	0.1	0.3	0.0	0.0
Oligochaeta	Oligochaeta	86.9	149.3	20.4	14.7	52.3	33.3	95.8	158.2	294.2	250.0
	Stylaria lacustris	0.0	0.0	0.1	0.1	4.3	4.7	0.4	0.9	2.4	2.2
Ostracoda	Ostracoda	0.0	0.0	0.4	0.6	29.6	28.0	0.0	0.0	0.2	0.2
Platelminty	*Dendrocoelum lacteum*	0.0	0.0	0.1	0.1	0.0	0.0	0.0	0.0	0.0	0.0
	Dugesia lugubis	0.0	0.0	0.1	0.1	0.0	0.0	0.0	0.0	0.6	0.7
	Planaria torva	0.0	0.0	0.1	0.1	0.0	0.0	0.0	0.0	0.4	0.8
	Polycelis hepta	0.0	0.0	0.1	0.3	0.0	0.0	0.1	0.3	0.1	0.3
Plecoptera	*Nemoura* sp.	3.5	5.5	0.1	0.3	0.0	0.0	0.1	0.1	0.0	0.0
Porifera	Porifera	0.0	0.0	0.0	0.0	0.1	0.1	0.1	0.1	0.0	0.0
[illegible]opte ra	*Adicella reducta*	0.0	0.0	0.2	0.2	0.0	0.0	0.0	0.0	0.0	0.0

Agrypnia sp.	0.0	0.0	0.0	0.0	0.1	0.1	0.1	0.1	0.0	0.0
Athripsodes sp.	0.1	0.3	0.1	0.3	0.0	0.0	3.1	6.1	0.0	0.0
Beraeodes minutus	8.5	17.0	7.7	15.4	0.3	0.5	0.1	0.3	0.0	0.0
Ecnomus tenellus	0.0	0.0	0.0	0.0	0.0	0.0	0.0	0.0	0.1	0.3
Glyphotaelius pellucidus	2.4	1.8	0.0	0.0	0.1	0.1	0.0	0.0	0.0	0.0
Halesus radiatus	0.0	0.0	0.1	0.3	0.0	0.0	0.0	0.0	0.0	0.0
Holocentropus sp.	0.0	0.0	0.2	0.2	0.0	0.0	0.0	0.0	0.0	0.0
Hydroptila sp.	0.0	0.0	0.0	0.0	0.0	0.0	0.0	0.0	0.3	0.6
Hydropsyche sp.	0.0	0.0	0.0	0.0	0.0	0.0	0.0	0.0	0.6	1.0
Limnephilus sp.	6.3	8.6	1.4	2.7	0.1	0.1	0.0	0.0	0.5	1.0
Mystacides sp.	0.1	0.3	0.1	0.1	0.0	0.0	0.0	0.0	1.6	3.3
Notidobia ciliaris	1.6	2.6	1.6	1.9	0.1	0.1	0.0	0.0	0.0	0.0
Oxyethira sp.	0.1	0.1	0.0	0.0	0.0	0.0	0.0	0.0	0.0	0.0
Phryganeidae	0.0	0.0	0.0	0.0	0.0	0.0	0.0	0.0	0.1	0.3
Plectrocnemia sp.	0.1	0.1	0.1	0.1	0.0	0.0	0.0	0.0	0.0	0.0
Polycentropidae	0.1	0.1	0.0	0.0	0.0	0.0	0.0	0.0	0.0	0.0
Potamophylax rotundipennes	3.4	4.7	0.0	0.0	0.0	0.0	0.0	0.0	0.0	0.0
Sericostoma personatum	0.1	0.1	0.0	0.0	0.0	0.0	0.0	0.0	0.0	0.0
Silo nigricornis	0.1	0.3	0.0	0.0	0.0	0.0	0.0	0.0	0.0	0.0
Triaenodes bicolor	0.0	0.0	0.0	0.0	0.0	0.0	0.4	0.9	0.1	0.1

Table S4: Indicator taxa per land use (Forest, EG – extensive grassland, IG – intensive grassland, cropland and WWTP – wastewater treatment plant) and respective p-values (package indicspecies; multipatt).

	forest	EG	IG	cropland	WWTP	stat	p.value
Micropsectra sp.	x					0.95	0.05
Pilaria sp.	x					0.87	0.01
Potamophylax rotundipennes	x					0.87	0.02
Glyphotaelius pellucidus	x					0.86	0.01
Corynoneura sp.	x					0.78	0.03
Apsectrotanypus trifascipennis	x		x			0.97	0.00
Paratendipes sp.	x		x			0.93	0.01
Hydracarina	x	x	x			0.98	0.01
Pisidium sp.	x	x	x	x		1.00	0.00
Gammarus sp.	x	x	x		x	0.96	0.05
Ostrachoda			x			0.99	0.00
Anisus sp.			x	x		0.88	0.05
Procladius sp.		x	x	x		0.97	0.01
Sialis lutaria		x	x	x		0.90	0.01
Valvata sp.			x	x	x	0.92	0.03
Stylaria lacustris			x		x	0.90	0.01
Chironomus sp.				x	x	0.97	0.04
Helobdella stagnalis					x	0.98	0.03
